# Therapeutics and Materia Medica

**Published:** 1860-07

**Authors:** 


ART. II.—*Therapeutics and Materia Medica; A Systematic Treatise on the Action and Uses of Medicinal Agents; Including their Description and History.* By ALFRED STILLÉ, M.D., late Professor of the Theory and Practice of Medicine in the Medical Department of Pennsylvania College, formerly Physician to St. Joseph's Hospital, President of the Pathological Society, and Fellow of the College of Physicians of Philadelphia, etc. etc. etc. Philadelphia: Blanchard & Lea. Two vols. 8vo., pp. 813–975.

A NUMBER of treatises on any given subject constitutes, of itself, no objection to an increase of them, provided the new work be marked, either by the introduction of fresh and pertinent facts, or the better elucidation of previously known principles; an improved distribution of the several matters discussed, or even a clearer description and narrative of what had already been known. Following, as the present work of Dr. Stillé does, the labors in the same field of three of his Philadelphia contemporaries—Drs. Dunglison, Thomas D. Mitchell, and Wood, who had been professors and teachers as well as writers on the subject, not to speak of the almost encyclopedic production of Pereira, of foreign origin—we had a right to expect something distinctive and additional, to entitle it to particular notice and consideration. This we believe it to possess on more than one of the grounds just specified. It is well written, its tone is calm and philosophical, and its matter copious and conveniently arranged, both for the purpose of scientific connection and easy reference. The author, while drawing freely from the medical literature of Europe as well as of his own country, has contrived to be learned without pedantry, and to give us the light derived from modern without neglecting ancient lore. In this expression of our opinion of the voluminous treatise of Dr. Stillé on therapeutics and materia medica as a whole, we do not, however, consider ourselves pledged to abstain from criticism on some of its parts. Where, indeed, is there a work of nearly eighteen hundred pages which is not open to some critical thrusts? Ours will be neither deep nor numerous; they will hardly go beyond, here and there, a slight rubefaction.

What to prescribe, how to prescribe, and when to prescribe, are the conditions for the successful treatment of disease, which we profess to cure by the aid of materia medica, pharmacy, and therapeutics. The first tells us what articles or substances are given to us for selection, after our learning their origin and intimate composition, and their effects on the organic tissues, and through these on the living body in general; the second (the how) teaches the modes of collecting, preparing, preserving, and compounding medicines, with a view to their most convenient administration; the last (the when) indicates the circumstances in the morbid state of the body which are to be changed, palliated, or remedied by agents of the materia medica, classified in reference to certain ascertained modes of operation on the several organs and vital properties of the living human body. If the order in which the parts of the title of a work are prefixed were to correspond with that of progress, from the simple to the more complex of the subjects treated of, we might ask that writers would, both in title and description, give materia medica precedence over therapeutics. Such was doubtless the natural and experimental course of observation and inquiry, in relation to the effects of medicines on the animal economy. But in the present, as in some other departments of science, in which results are described without detailing the steps by which they have been reached, the synthetical is made to prevail over the analytical. In studying history, for example, the writer gives his own narrative and views, and the conclusions which he has drawn, and refers his readers to documentary matter, in notes and appendix, for proofs of his accuracy and the correctness of his judgment. We will not, therefore, make any objection to the title of Dr. Stillé's work—for which, by the way, he has good precedents—but proceed to notice some of its chief features, which, if not original, have at least a pleasant air of freshness about them, inviting to further and more familiar acquaintance.

The "Introduction," true to its title, does really prepare us, step by step, for a further and detailed view of the subjects which are successively brought to our notice as we advance.

"Works on therapeutics are complementary to works on practice of medicine. In the latter, many remedies are examined from a point of view in a single disease; in the former, many diseases are examined in their relations to a single remedy. Either would be incomplete without the other; and he is the best practitioner of medicine who most certainly and clearly keeps before his mind the reciprocal dependence of the two great departments of the art of healing."

In the subsection, on the "*Sources from which Medicines are derived and the Causes which modify their Virtues*," occurs the following remark, which we have long been in the habit of making when speaking of quinia and its salts, as the alleged representatives of the Peruvian bark: "In nature a medicine seldom exists in a pure or isolated state; and although chemistry has extracted what are called the active principles of many drugs, as quinia and its salts from cinchona, and morphia from opium, it has at the same time revealed, that nature has associated with these principles certain others which, subordinate though they may be, are probably essential to the effects produced by the drug in its natural state." Bearing in mind these facts, we shall have recourse, in obstinate cases of periodical fever, to the bark in substance and its officinal preparations in addition to quinia; and when we are baffled in procuring sleep for our patient by the salts of morphia, we shall fall back on opium in substance, or in combination with ipecacuanha and a neutral salt, in that never-to-be-too-much-praised Dover's powder.

"On the Sources of Knowledge in Therapeutics," the author, among other points, presents the following. He had just been speaking of the instinctive cravings manifested in disease, as suggesting the use of certain remedial means:—

"Every one has witnessed the intense desire for cold drinks usual in acute febrile diseases, the craving for acids in scorbutic affections, and in some others distinguished by a diminished crasis of the blood, and also the longing for innumerable and widely dissimilar articles of food, many of them disgusting, or in themselves unwholesome, by pregnant females. The chlorotic girl who greedily devours plaster, and chalk, and charcoal, obeys an internal craving, which is often explained by an excessive secretion of an acid, which these substances absorb; and, as Albers has remarked, the selection which she will sometimes make of such articles is wonderfully fastidious. He met with children who relished charcoal made from boxwood, but not from oak; soft but not hard chalk, old but not new plaster; and we remember a young lady, of marked chlorotic aspect, whose unnatural appetite could only be satisfied by a certain ferruginous pebble, which she diligently searched for in the gravel-walk of her garden."

It is mortifying to reflect, how often instinctive cravings have been determinately opposed by physicians, under the influence of speculative notions, as in the familiar instance of the desire for cold drink in the exacerbations of fever. In place of gratifying the patient in his intense longings, he was told to use warm drinks instead; or in other cases, as in dropsy, he was subjected to the horrors of thirst. So, also, in the morbid processes going on in the intimate structure of the organs, which, left to themselves, would often end in restoration of healthy tissue and organic action, but which, interfered with and interrupted by art misapplied under the directions of false reasoning, pass into further and destructive changes. More obvious suggestions for the relief of suffering and disease were furnished in the effects of accidental and temporary increase of some functional actions, as in purging, sweating, and diuresis, and even hemorrhage; and the inversion of others, as in vomiting. Attempts to imitate these evacuations "were doubtless made at first, and derived by imitation from the lower animals, or by an accidental experience of the powers of different agents on man."

"Until the time of Galen, who lived in the second century of the Christian era, medicine hardly claimed the rank of a science—or a philosophy, as it was then termed—but contented itself with a diligent observation of nature and the results of experience. These were gathered and observed by Hippocrates, Theophrastus, Celsus, Dioscorides, and Aretæus, and have continued to be the fountain from which later ages, even to the present time, have drawn their chief supply of therapeutical knowledge."

The attempt of Galen to frame a system of medicine in which practical precepts were deduced, not from experimental observation but from previous speculative dogmas, was successful in securing support from his followers for thirteen centuries, but it utterly failed when subjected to the tests of experience and induction.

"Before proceeding to consider more particularly the sources from which our knowledge of the curative power of medicines is derived, it is proper to premise what should be understood by the phrase, to *cure* disease. The word to cure, in its proper and etymological sense, means 'to take care of,' and it is only derivatively that it has come to signify to heal or 'to restore to a sound or healthy state.' The distinction is happily expressed in the Latin line, *Medicus curat, Natura sanat morbos*—Physicians cure, but Nature heals. Medicine is only the handmaid of Nature, the really active and efficient agent in the restoration of health; medicine can do no more than remove the impediments from Nature's path, support her when faint, restrain her when violent, and guide her when she is inclined to err. But the vital powers and functions of the organism have an inherent tendency to return to their normal condition when deranged by any cause, and to remove or repair the alterations of structure which may have attended that derangement. In this consists the healing power of Nature, *vis medicatrix Naturæ*, which, under various names, was recognized even in the earliest ages of medicine, and indeed more fully then than at any subsequent period until modern times."

We regret that Dr. Stillé should repeat, both in the above and succeeding paragraphs, these commonplaces of metaphorical ascription to Nature, as to an efficient cause, the production of a series of phenomena which are, in truth, nothing more than a series of antecedents, and in some instances of concurrents, with the real cause of which we are unacquainted. Nature, as here used, like the *animus* of Stahl, the *archæus* of Van Helmont, and the *vital principle* of Barthez, is a mere metaphysical ontology, a speculative abstraction, which has not even the secondary merit of elucidating or explaining anything. We may say of it what Comte says of the vital principle of Barthez, that it assumes to be a real and complicated existence which is profoundly unintelligible. It was with similar language, as for instance functions and occult virtues, that chemistry and physics were loaded and their progress retarded during their metaphysical period. The ordinary language, the course of nature, the laws of nature, etc., is never thought to imply a causative or governing power in an entity called nature, but merely the expression of a certain uniformity of progress and regularity of results ascertained by sufficient induction. The successive series of natural phenomena cannot be understood, certainly cannot be described by an abstraction, in our assuming the presence of an imaginary existence—spirit, soul, or nature—to produce these results. The contrast between the figurative and the descriptive, the unmeaning and the meaning, is well exhibited by the author in two sentences; the one following the other in the second of the paragraphs which have furnished mätter for these comments of ours—"The highest achievement of art is to sustain nature, that she may have time if possible to perfect her work." Here are two metaphysical entities invoked to perform important parts in the field of therapeutics, but what they actually do is beyond conjecture even, and must be left for imagination to disport itself on. What a contrast to this poetry of the subject is offered in the very next consecutive sentence! "The whole process by which wounds are healed is a natural one; and consists simply in the establishment of the nutritive process between the divided surfaces; of the exudation of a plasma, and of cells; the formation of blood-vessels; and the absorption of the excess of the material." If immediately after this he indulges in the redundant phraseology of "this simple method of nature," and of "nature" as "so efficient in healing external lesions," we need the less object, as the previous descriptive text is, of itself, clear and comprehensible. Is the following sentence rendered more effective in causative assumption—certainly it is not in description—by the words "referring to nature"? "Even in ordinary inflammation, the tendency to form an abscess, or at least to erect a barrier of coagulated albumen between the central seat of the lesion and the surrounding structures, illustrates the conservative power of nature." Would it not be somewhat more expressive of that which actually occurs on this occasion, to refer it to the inherent conservative power of the living organism—a property of the material parts in action, a sequence of antecedent phenomena of living structure, but not a *Bona Dea*, an imaginary creative-spirit? Believing the author to lean, if he does not adhere to the positive philosophy of Comte, we have been the more free to comment on his retaining some of the metaphysical subtleties of the olden time which are discarded by that philosophy, and which are opposed to the prevailing manner of both physiological and pathological investigation at the present day. There are still medical as well as other superstitions which obstruct progress in this age of boasted enlightenment and free inquiry.

Reference is made by Dr. Stillé to "the doctrine of crises, which formed so capital an element of the Hippocratic pathology, and the one of all others which does not countenance a perturbative treatment." An apology is offered for the inability of physicians to save the lives of the sick, on the ground of there being in some cases a destruction of organ, which precludes the possibility of restitution under any conceivable circumstance; in others, a vitiation of the blood, which cannot be readily ascertained, nor, when known, easily corrected. Between the two extreme classes—of diseases cured, as he terms it, by nature, and of those in which neither nature nor art avails to avert death—"there is," the author adds, "a middle class in which the skill of the physician and the power of his art are chiefly manifested. They are affections, some of which have little or no spontaneous tendency to cure, as goitre, syphilis, periodical malarial diseases, etc., or else incidental states or phases of continued fevers, inflammations, and other more specific affections."

The next topic discussed is on the "Sources of Knowledge respecting the Action of Medicines." While affirming, as "a fundamental truth, that therapeutics must be learned at the bedside of the sick," the author points out the difficulties in the way of determining the essential action of the medicine employed, and the common and special embarrassment in distinguishing between the symptoms of a disease and the effects of a medicine. Clinical observation is, in a peculiar manner, liable to fallacies of this nature, and often the practitioner complacently attributes to his prescriptions effects which belong to the successive changes and stages of a fever, or of an inflammation. From the pride of opinion thus engendered, "the transition is short and easy to a positive and willful misrepresentation of truth, to creating facts to sustain a theory, or to alleging results of treatment different from the true ones, perhaps the reverse of them entirely." New medicines are introduced almost every year to the exclusion of old ones, whose sole recommendation, it must be confessed, is in some instances the length of time in which faith has been put in their efficacy. A more rigid inquiry in a better method of investigation, has, "it is true, not only permitted many of the ancient remedies to maintain their place with undiminished reputation, but has even strengthened it by revealing and adding to the grounds upon which it rests. It has also enriched the healing art with a great number of new remedies, whose value has been tested and confirmed by observations carefully made in many countries, and under varying circumstances during a long series of years."

The author advances a brief defense of the numerical method, as applied to therapeutics:—

"Doubtless there are elementary conditions or lesions, however occurring, which are uniformly controlled by the same remedy; depletion everywhere modifies, at least, the forming stages of inflammation; mercury everywhere acts upon its products, opium in all cases assuages pain, etc. These uniform effects are determined by observation and the aggregation or addition of similar facts; and the more systematically they are ascertained, the more absolute will the results become. The latter class of general facts form the foundations of general therapeutics, the former the laws of special therapeutics."

Complaint is very justly made of continual trespass on the domain of therapeutics by pathology, physiology, and chemistry. Their ancillary aid cannot be refused; but their arbitrary assumption of lead and of abilty to solve the recondite problem of the cure of disease are matters for grave challenge and rebuke of inadmissible pretensions—we have, in former years, sometimes called them impertinent intrusions. The author, pursuing his argument of the nullity of *à priori* observation or research, says that there is not, he believes, "a single example of a medicine having been received permanently into the materia medica upon the sole ground of its physical, chemical, or physiological properties." It would not have been amiss, at this point, for him to admit of some qualification of his adverse opinions, in favor of the suggestions which botany sometimes furnishes of the probable medicinal activity of a plant, seen for the first time, from the fact of its belonging to a great natural family, the *convolvulaceæ*, the *umbelliferæ*, or the *solanæ*, for example. The accordance between the properties of plants and their physiognomy is not, however, of such uniformity as to furnish material for more than suggestion, when we know that among the convolvulaceæ we find jalap and scammony, and that from the same family comes the sweet potato; and if the unbelliferæ yield cicuta, so do they, also, our common parsley. The *cucuritaceæ* yield the drastic colocynth and elaterium, and give us, notwithstanding, the melon, the gourd, and the equivocal cucumber. The exception to the last examples is perhaps more apparent than real, for a bitter principle is found in the rind of the melon and cucumber analogous to elaterium and colocynth.

After speaking of the confirmation of the results of clinical observation furnished by experiments upon the healthy organism in man and the lower animals, he defines the uniform action of medicine upon healthy structure or function to be its physiological operation; and its therapeutical to consist in its curative action upon diseased structure or function. "But the latter involves infinite difficulty; for we are required to determine the influence of an agent upon functional and structural conditions, with the natural terminations and tendencies of which we are only imperfectly or not at all acquainted." With all its imperfections, however, we must take, as the only available standard, the operation upon the healthy body of the same medicines which we administer in disease.

"But let us not suppose, that even this method is exempt from difficulties and imperfections. The perfectly healthy constitution which the problem demands is seldom to be found; or else individual peculiarities exist which, unless the experiments are frequently repeated, require us to accept their results with circumspection. Age, sex, and constitution modify the action of medicine, and accidental circumstances, such as climate, season, and occupation, are not without their influence. To take a single example: the doses of cathartic medicines which are habitually used in cold and temperate regions would be fatal in tropical climates. Idiosyncrasies, as they are called, peculiar and inextricable susceptibilities, frequently modify and even reverse the ordinary action of a drug. Ipecacuanha in the smallest dose may occasion a violent coryza, or the least quantity of mercury induce ptyalism or produce nervous prostration; opium may induce wakefulness instead of sleep, etc."

"We can with some confidence foresee the effects of medicines in health, but in disease there is no experienced physician who does not feel uncertainty, and indulge in some self-gratulation when the result corresponds with his predictions."

"There is a large amount of information, respecting the operation of medicines, to be obtained from a study of a case in which they have been taken in excessive doses by mistake or with criminal intent."

Experiments on animals are declared to be "of great value, because they enable us to make a large number of comparative observations, varying the conditions at pleasure, so as to arrive, by exclusion, at those effects which are peculiar and essential to each medicine. We can also observe the different modes of their action, according as they are administered by the stomach or the bowels, applied endermically or to the surface of wounds, injected into serous cavities, into the blood-vessels, etc. Further, we can in this manner learn the diversity of effects produced by various doses, degrees of dilution or division, and modes of combination of the several medicines. But more distinctly than in any other way we can thus discern the lesions which are produced in the body by medicines capable of acting as poisons, according to their dose, form, etc."

It is to be remembered, in this place, "that many medicines do not affect man and the lower animals alike. They may be poisons to the former, and food to the latter. Hemlock and water-hemlock (*conium maculatum* and *cicuta virosa*) are eaten in considerable quantities by cows without injury, but they are poisonous to man. Albers relates that he gave ten grains of pure morphia to a rabbit without producing any effects of opium. To another he gave twenty-five grains of this substance without destroying it. Eight days afterwards he administered to the same animal a drachm of opium and ten grains of morphia. It lived, and gave no sign whatever of narcotic poisoning. Horses can take large doses of arsenic without injury—so large indeed, says Albers, 'that they would destroy as many men as would weigh twice as much as a horse.' Infusion of quassia, which to man is a salutary tonic medicine, is extremely poisonous to flies, and destroys even dogs when injected into the veins. In herbivorous animals, as the rabbit, vomiting never occurs; while in carnivorous animals, as the dog, it is readily excited. Moiroud states that colocynth, jalap, gamboge, and bryony, which operate as violent purgatives on man and carnivorous animals, have comparatively little effect on the horse and other herbivorous animals."

The copious citation of facts, to strengthen the propositions laid down, and arguments advanced in enforcement of principles, give peculiar value to Dr. Stillé's work, and tempt us, at every moment, to make extracts from its pages. We must, we find, resist this temptation, as it would carry us far beyond any reasonable limits of analytical review,—although our readers would easily excuse us for each instance of excess in this way, for the additional instruction received by them on the occasion. The section on the "Physiological Action of Medicines," in its two divisions of the *local action of medicines* and their *remote action*, is very rich in experimental matters to illustrate the questions which come up on the effects of medicinal agents, in virtue of their penetration and absorption, and through nervous agency and the circulation of the blood. We regret much not being able to quote at large the interesting summary, drawn up by the author, of the various experiments, and deductions from them, on absorption, made by different individuals within the last half century. We may say the same of the section on the "Conditions for the Absorption of Medicines," and the "Avenues by which Medicines are introduced and the Various Effects which they Produce"—under the several heads of "*Infusion, or the Injection of Medicines into the Veins; the Stomach; the Rectum; the Mouth and Fauces; the Skin; Inhalation.*" On the subject of endermic medication many valuable experiments and observations are collected and collated. Those on the method of introducing medicines through the skin by inoculation are particularly fresh, and suggestive of much improvement in our therapeutical resources. "The Changes which Medicines undergo after Absorption," is a theme of great practical moment, ably discussed.

The subject of the "Curative Action of Medicines" opens a wide field for inquiry, which is carried on under the heads of the *primary* and the *secondary* operation; sometimes one and sometimes the other of which is curative. The spécific action of medicines requires, and has obtained from the author, a particular examination. We are not to suppose that in using the word specific, any intelligent physician means to say that there is any substance thus designated which will cure a disease infallibly and universally, or work like a charm; but that its general effect is to remove a disease without its producing any marked change in the sensations or functions of the organism, as in the instances of cinchona curing intermittent fever, iodine goitre, etc. "Some medicines tend to escape from the body through certain organs rather than through others, and in doing so, to exert a stimulant influence upon them, or, in addition, as in the cure of certain glands, to modify their secretions." A detailed statement is given of the several organs and the medicines which manifest an elective affinity for them. According with what had been learned from long previous clinical experience, we are told that "of medicines that act upon the *liver*, mercury is the chief." In experiments upon animals, "the secretion of bile was augmented by the administration of mercury, and this substance has afterwards been found in the hepatic duct. Oil of turpentine renders the bile thinner and more abundant; coffee, on the contrary, makes it thicker and darker; alcohol increases its fatty constituents and those of the liver. Sulphates and tartrates are said to be cholagogues, and so is aloes; but although the experiments of Jorg appear to favor this view of the action of aloetic purgatives, it is not generally adopted; the coloring matter of the drug, it is thought, has been mistaken for that of the bile."

"Sedatives of the *heart* and *blood-vessels* include digitalis, white and green hellebore, aconite, colchicum, and squill; the principal stimulants of the same organs are alcohol, wine, iron, and assafœtida." One of the medical creeds of the day would seem to be supported by no adequate evidence, if we take the following as indications of the facts of the case: "Albers states that phosphoric acid, which in old and in recent publications is alleged to have a specific operation upon scrofulous and rachitic affections of the bones, he has found, by repeated trials, to be wholly destitute of such a virtue."

"Influences Modifying the Effects of Medicines" are discussed with due method and precision, under the heads of "*Diversities in Medicines themselves,*" and "*Diversities presented by Patients; Influences of Race, Climate, Season,*" etc. Climate, season, locality, and soil exert, severally and in combination, a strong modifying influence on the activity of the medicines from the vegetable kingdom. "The hemp of Northern Europe and of the United States possesses no remarkable medicinal qualities, while that of Asia (*Cannabis Indica*) exudes a juice which is a powerful narcotic. All extracts made from the roots and herbs of plants are comparatively worthless, if obtained when the juices are diluted and feeble in the spring." The form of a medicine, its dose, full or small, or in divided doses, are important qualifying conditions not only for its greater or less activity, but for its operation and curative effects. Tartar emetic, for instance, in a dose of a grain diluted in water, acts as an emetic of considerable power. In smaller fractional doses, repeated at intervals, it reduces the pain and abates all the phenomena of fever and inflammation. The same quantity of a medicine, calomel for example, taken at once or spread over a period of time in divided doses, will be followed by a totally different series of actions, both physiological and therapeutical: the most prominent points of contrast will be found in its freely purging in the first instance, and its salivating in the second. "The doses of medicine vary with the parts of the body to which they are applied—the stomach, the sound skin, the rectum and colon (by enema,) the eye, endermic application, ulcers and suppurating sores."

Dr. Stillé has omitted to refer to them, although he must be well aware of the facts of the modifying influence of the time of the day and the period in a disease in which medicines are administered, on their direct effects and therapeutical value. All medicines taken by the mouth in the morning will be found to exert a speedier impression not only on the gastric and intestinal surfaces to which they are applied, but, also, on remoter organs which are acted on by the absorption of the medicine and the circulation or by nervous transmission, than in a later hour of the day. We turn this knowledge to useful account in prescribing a purgative in the morning, or, to meet a different indication, a tonic or a diffusible stimulant. There is an accumulated excitability after a night's sleep, which causes the organism to respond with increased readiness to every kind of stimulus applied to it. Very different also will be the effects of the several agents specified according as they are given in the period of the remission or of the exacerbation of a fever. A purgative taken at the former will act well and fully; at the latter, it will increase the febrile irritation, and probably fail entirely to produce its ordinary first and sensible effects. A tonic in the morning remission will often prevent the recurrence of the febrile paroxysm; given when this state has supervened, it will aggravate its worst features and prolong their duration. So, *à fortiori*, in the case of diffusible stimulants given at these different periods. Narcotics, as hypnotics and anodynes, may, on the other hand, be expected to produce their desired soothing effects in the evening, when the body has been weakened and to a degree exhausted by the excitement, and, in chronic cases, the exercises and labors of the day, and, in consequence, requires less extraneous or medicinal assistance to produce the expected and coveted sleep. If the physician has the day at his disposal, he will, therefore, imitating to a certain extent the rhythm of the healthy functions, direct, if the case requires such medication, a purgative early in the morning and a hypnotic in the evening.

There is yet another point coming under the present head which demands long and patient observations, and a series of experiments far beyond what it has yet received. It is, the period of the entire and continuous operation of a medicine, taken into the stomach, on the organism. Ignorant of the successive stages, and of the duration of each of them, we cannot tell whether the article we prescribe at the moment may not be subjected to a decidedly modifying influence by the yet persisting operation of another and contrasted agent administered some hours previously. Our notions on this matter are sufficiently crude and limited; as, for example, when we speak of the operation of an emetic or a purgative being over, after the evacuations from the stomach or the bowels have ceased—and deem ourselves free, in consequence, to prescribe forthwith another medicine to meet a new and different indication. Careful observation would show that the active effects, as we may term them—and by this we mean more than mere emesis and purging, will last, or perhaps we should say continue to be developed, during the whole diurnal period; and if so, these effects must be interfered with, and even combated or neutralized, by another kind of medication, or they will, themselves, weaken or counteract the results which we anticipated from this latter, if given within this period. Would an additional diurnal period be too long for a purgative to produce the full series of its effects, including the quieting, almost sedative ones which succeed the revulsive, as these did the evacuating, which in their turn followed the primary irritating operation of the medicine? Must not an excitement, bordering on the febrile, which follows the first and even second series of actions caused by a purgative, have subsided before the nutritive system can resume or regain, as the case may be, its customary functional activity so as to allow of the full enjoyment of alimentation and of the full vigor of subsequent digestion, chylosis, and hematosis? With our attention fixed on the successive series of phenomena thus produced, and which each person can observe on himself, in ordinary circumstances, in which a purgative is taken to correct a slight indisposition or ailment, we can approach to a consideration of the more complex problem of its full operation in a fixed and febrile disease.

The difficulty of determining, with more than a bare approximation, the time occupied by the successive stages of the operation of a narcotic, whether it be hypnotic, anodyne, or mixed sedative and stimulant, is continually felt by the medical practitioner. A spirit of routine prevents him from appreciating the fact of the time that must generally elapse between the ingestion of the medicine and its first anticipated therapeutic effect, and the often longer time between the subsidence of the anodyne and soporific operation and its subsequent disturbing and depressing ones. How common to prescribe an opiate, with a view to its procuring sleep, at the ordinary hour of retiring in the evening, or at bedtime, as we call it, and to learn, in the morning, that the coveted sleep did not visit the patient until the morning was come, and with it sounds and distracting causes, in the house and out of the house, which rouse him from slumbers but half enjoyed! And yet the inference is still often lost sight of, viz., the propriety of prescribing the opiate at an earlier hour in the evening, and allowing the patient longer time for its prolonged effect in the morning. An analysis of the more obvious secondary effects of narcotics, after the anodyne and soporific ones have subsided, would probably show that they continue during nearly the whole of the following day; and that if we prescribe other medicines during this time, it ought to be with a perception of an increasingly complex problem before us, made up of the disease we are treating, the continued effects of the medicine or combination last used, and those of the substance now given with a view to its immediate curative action. To causes of this nature must we attribute the complicated features, the anomalous symptoms, which embarrass the physician in the more advanced stage of the disease. If he have self-possession and firmness he will endeavor to simplify the problem, and to reduce it to fewer elements, by withholding all medicines for a certain period, it may be a day or more. He will then be able to discover what was due to the disease itself, what to prior medication.

Under the head of diversities produced by patients, reference is made to *age, sex, habit, idiosyncrasies, and mental influences,* and appropriate remarks are made on each of these modifiers. In speaking of the influence of race, climate, season, etc., the author remarks: "It is difficult, in reference to the present subject, to separate the influence of race and climate, for it is generally found that the foreigner who is acclimated and has also adopted the mode of life peculiar to the natives of his new residence acquires a similar susceptibility to the action of medicines." This is too limited a view of the question, which ought to embrace the therapeutical differences of susceptibility between distinct races, the Caucasian, the Mongolic, and the African in their respective countries, as well as a comparison between two of them, the white and the black for example, in the United .States, under similar circumstances of climate and locality, as also of the white and the yellow as far as we find them together in China, and two great families of the same race, the Hindoo and the Anglo-Saxon, as they present themselves under the same climatic influences in Hindostan. Colonization, commerce, and travel place it in the power of intelligent observers to extend and give some fixedness to our knowledge in this matter, of which in earlier times the world would have remained hopelessly ignorant. Dr. Rush, in one of the "Inquiries," which we noticed with some fullness in the last number of the REVIEW, broaches the question, by reference to the dietetic and medicinal usages of the Indians of North America compared with the civilized people of the white race. The therapeutical and surgical records of Dr. Parker and other medical missionaries in China, leave the inference that the people of that country bear up after injuries and operations to an extent rarely seen among Europeans or Anglo-Americans.

The cautions by the author to have recourse to depletion and the use of active medicines with great reserve in old age, are in the main correct; but they admit of considerable qualification, and must not be received to the extent of withholding depletion by blood-letting and purgatives, in cases in which the symptoms would point beyond dispute to their use in younger subjects. We have, ourselves, used venesection in hæmoptysis, pneumonia, and enteritis, in persons of both sexes who were past their seventieth year, and with effects which, considering the recovery of those persons and the subsequent comfort and average health they enjoyed, were, to say the least, far from regretable. In every instance, however, this active medication was only had recourse to after the obvious failure of milder means to procure even an amendment of the violence of the disease.

There is yet another means of modifying the action of medicines when used for therapeutic purposes, which, although often neglected, is of the highest importance. We refer to the increased tolerance and renewed susceptibility of an organ or organic apparatus, to an article of the materia medica or a pharmaceutical compound in consequence of the operation of a previous remedy. How often in the early stage of fever, not to speak of distinct gastritis, is the stomach in such an irritable state as to be unable to retain a purgative or even a mild laxative, and how often is the trial repeated to procure evacuation from the bowels by giving a succession of medicines whose operation would, it is hoped, bring on this result. Each attempt in this way is only a fresh source of irritation, until the stomach, if it could become vocal, would entreat for rest, which, it must be confessed, is of itself sometimes adequate to the desired end. But, more readily and confidently, shall we be able to tranquilize the stomach and enable it to retain the purgative, by prior recourse to venesection, and to the application of leeches over the epigastrium, and sometimes of a remedy of an entirely different class—a vesicatory or a sinapism?

After a patient has been subjected to the double disturbance of his organism, by the recurring paroxysms of periodical fever and a succession of stimulants and tonics, from quinia to centaury and chamomile, including all the changes to be rung on the first of these articles, in augmented doses and in various combinations, without benefit, a change in the susceptibility of the stomach and nervous system by remedies of a different class, as by a single venesection or a few grains of calomel or even blue mass, will make the entire organism amenable to the full anti-periodic influence of the very same articles, in the very same doses, and forms of combination, which had previously failed to produce any curative effect. So also will the desired soothing and soporific effects of opium be more certainly obtained after venesection, or the operation of a purgative.

Dr. Stillé's rich introduction to his treatise ends with some advice and details on the "Administration of Medicines," under the subheads of *The Forms in which Medicines are employed*, the *Art of Prescribing*, and finally with a separate section on the "Classification of Medicines." The author has twelve classes; the eleventh being evacuants. These are epispastics, errhines, sialagogues, emetics, cathartics, expectorants, diaphoretics, diuretics, emmenagogues, anthelmintics. The twelfth class consists of alteratives. In the difficulty of finding any natural classification, we must still always admit the necessity of making one in this as in other departments of science, and especially in natural history. A classification is an aid-memory, and prevents repetitions which would become wearisome.

We shall not attempt to follow Dr. Stillé in his accounts of the *modus operandi* and general indications for the use of the several classes of medicines; and still less can it be expected of us to notice even the prominent articles of each class. In skimming over his fertile pages, we shall merely stop here and there for a moment to cull, it may be to scrutinize, some of their multifarious products.

*Bitter almonds*, placed among the first class, or lenitives, may be used in the same cases in which hydrocyanic acid is administered. "In such cases the essential oil may be prescribed in doses of one-quarter of a drop to a drop, in emulsion. But when a demulcent as well as a sedative operation is required, the emulsion of bitter almonds is to be preferred."

In catarrhal affections of the mucous membranes, it is both a pleasant and a useful preparation. The simple almond emulsion has long been used to remove sunburn and freckles.—*Glycerin*, in the proportion of one part to five of flaxseed tea as enema, is an excellent remedy in dysentery, by soothing the irritated rectum and suspending the tenesmus. It is well spoken of as a local application to the interior of the larynx in membranous croup. In partial loss of the tympanum and in epithelial thickening of the meatus, glycerin introduced in the external meatus has been productive of much good. It exerts a favorable action on wounds, ulcers, and burns, and, combined with tannin, in vaginal leucorrhœa by means of a solution of camphor saturated with one part of the former to four of the latter. This last combination in the proportion of one to sixteen, in solution, has also been used advantageously in fissures of the tongue and anus, and on chapped and fissured nipples. As a palliative it is useful, but only to this extent, in chronic diseases of the skin.—*Collodion*, like glycerin, is a new remedy. It was at first and is still employed as a substitute for adhesive plaster in the treatment of wounds; and subsequently and with great advantage in that of ulcers, especially when they are exposed to continued irritation, as in those of the neck of the uterus. It is a good preservative of slight wounds or abrasions from infections in dissection, surgical operations, and the practice of midwifery; it serves also to arrest excessive bleeding from leech-bites. In erysipelas, depending more particularly on local causes, this article has been applied with the best effects, and also in superficial burns. Collodion has not maintained its first reputation "as an ecbolic remedy in small-pox, to prevent the development of pustules upon the face and the unsightly scars which too often follow them." It has been used with more or less advantage in various diseases of the skin, as intertrigo, varieties of herpes, lichen, lupus, acne, chronic erythema, etc. In swelled testicle and in varicocele, collodion, by its constrictive effects, has been very useful. It should be first applied to the scrotum, near the root of the penis, and gradually extended downward, and somewhat to the opposite side.

"Böcker, who has carefully examined the question, shows that sugar is not directly a fat-producing substance, and that it cannot possibly contribute anything to the nitrogenous tissues." "The appetite for it displayed by children appears to be a natural instinct, the object of which is to enable the system to appropriate for the growth of the organs azotized food which would otherwise be consumed in the production of animal heat." The author inclines to the belief expressed by Böcker and Larrey, that sugar is injurious to the teeth. The expectations entertained from some trials made, of the beneficial and even specific agency of sugar as a cure for diabetes, have not been borne out by experience.

The following remarks on "astringents" are discriminative:—

"Each of the several astringents has powers which are peculiar to itself, and they can by no means be substituted for one another, so as to produce identical effects. Nitrate of silver and sulphate of copper excel vegetable astringents in consolidating ulcerated tissues, but preparations of tannin, or rather of some of the astringent barks, are far superior to those mineral remedies in preventing that softening of tissue which tends to terminate in ulceration. The first-named articles are also better adapted, as a general rule, for internal administration. Creosote, nitrate of silver, nitric acid, and other strong acids are more efficacious as styptics than tannic acid. Except creosote, these are also caustics. Several astringents possess likewise specific powers of a totally different nature. Lead, for example, in some of its forms, has a special aversion to the nervous system; and in another, constitutes a powerful deodorizer. Iron, which in certain of its combinations—as the perchloride—is eminently a styptic, is a tonic in its general operation. The sulphates of alumina, copper, and zinc are emetic as well as astringent, and the second is supposed to have some periodic influence, while the last is employed in the treatment of neuralgia."

Alum, in its primary local action, is decidedly astringent, and on this account it is productive of constipation; but if its action be sustained and prolonged it may ultimately increase the secretion of the part in place of diminishing it. In one of the most obstinate forms of constipation, that which depends on lead poisoning, it is used to overcome this state. "The familiar facts of practical medicine render it certain that when alum is taken into the stomach it is absorbed, and modifies the blood, and probably also the contractility of the animal fibres. But it is also found in the urine of persons who use it, and in the liver, spleen, and other organs of animals to which it has been given in large quantities." In all the experiments on animals to determine the effects of alum on the tissues and functions, the mucous membrane of the stomach was strongly inflamed, and in some cases erosions existed. "A drachm is usually sufficient to produce vomiting in a child under five years of age, but much larger doses have been taken as a remedy for intermittent fever, or otherwise, by adults, without any other effect than transient nausea." In lead colic the dose of powdered alum is from one to three drachms a day, given in solution. It has also been found very serviceable in cases of habitual constipation, producing or produced by atony of the bowels. In the doses of one or two scruples, Mr. Aldridge, quoted by the author, found that it would often produce large and very solid evacuations. "Associated with sulphate of magnesia, it corrects the unpleasant taste of this salt, and its tendency to produce flatulence."

A statement in the work before us, of the therapeutical and toxical operation of lead on the economy, reminds us of an opinion which we have long held, that this class of astringents is far from a natural one.

The several articles of which it consists are not by any means distinguished by close analogies to each other, nor by any harmony of action. What resemblance, for example, is there between the constringing of tissue and augmented tone of an organ, produced by vegetable astringents as represented by tannic acid, and the sedative and deadening operation of lead, running into anæsthesia and paralysis? Nor is there an approach to unity of therapeutical action if we look at diseases and their stages in which we prescribe the two agents. Preparations of lead are used in the very beginning of cases of inflammatory action, and in active hemorrhages; vegetable astringents in the decline of inflammation, if at all in this morbid state; and in passive hemorrhages followed by anæmia and great nervous debility; also in atonic dyspepsia. In the profluviæ, in which both are used, lead is chiefly beneficial by its sedative action, in allaying irritation and nervous excitement; tannic acid, the representative of vegetable astringents, does good by its increasing the activity of the nervous system and raising the powers of the economy.

Tannic acid, in addition to its now well-known efficacy in passive hemorrhages and in chronic mucous discharges, when taken internally, is also used locally with the best effects.

"As an external application to the skin, when an astringent remedy is required, Mr. Alison thinks it is of special excellence. He used an ointment of it with remarkable benefit as an application to psoriasis. But it can of course be only subsidiary to other means. It is of use also in preventing local perspirations, and particularly fetid discharges from the armpits and feet."

In these last-mentioned circumstances, the physician will not attempt a sudden suppression of the habitual discharge by purely local means, without the previous use of purgatives and alteratives. Gallic acid is highly praised in hæmaturia, and in purpura hemorrhagica, and has done good service in the relief of albuminuria.

In the chapter on "Irritants," the reader will derive instruction from the author's descriptions of the *mechanism of medicinal irritation*, and of inflammation. The *theory of counter-irritation* also is explained with considerable fullness, and an explanation offered of what are vaguely called sympathetic influences. Among *the elements of the counter-irritant action* pain ranks foremost, and *substitution* as a method of cure is suitably examined. Stimulant applications, made directly to an inflamed tissue for the purpose of curing it, are of very frequent use. This treatment "is applicable almost entirely to primary idiopathic inflammations." Counter-irritation, as revulsive according to the nature and stage of the disease, and the extent of a diseased part, as also the constitution of the patient, is described and explained. The depletory action of irritants is next inquired into, and suitable cautions are given not to have recourse to excutories without a strong conviction of their necessity, and not to discontinue their use without the creation of some supplementary evacuation by depletion, purging, or diuresis, or else by hygienic means adapted to prevent the undue production of blood, and especially its production in particular organs.

With whatever favor we may regard the views of the author on the philosophy of the general therapeutic action of irritants and of counter-irritation, we must, however, express our dissent from his enumeration of particular irritants, which, in different instances, is neither in harmony with his definition of the class nor with the acknowledged *modus operandi* and curative effects of some of them. His division of irritants into *rubefacients*, *epispastics*, and *caustics* or *escharotics*, and his description of their immediate effects, are suggestive only of topical remedies and local action externally applied; but, with few exceptions, they fail to give intimation of the constitutional operation and alterations in the functions produced by the internal administration and general diffusion through the organism of other agents which he calls irritants, but which make no approaches to being a rubefacient, a vesicatory, or a caustic. It would have facilitated an understanding, or at least a clearer idea of the subject, if the author had described the pathological state called irritation, for, in fact, under nearly every aspect in which we may regard it, irritation is either a primary morbid process or the imitation of one by certain agents of the class above described. We were for a moment tantalized by the words "mechanism of medicinal irritation," but in place of defining this process, the author passes on, at once, to speak of inflammation, from the successive phenomena of which he deduces the indications for the employment of counter-irritants. Failing to explain irritation as a morbid process, he also soon loses sight of it as an artificial condition induced by certain agents with a view to their curative effects, and he speaks, instead, of artificial inflammations palliating or curing those which arise primarily and idiopathically. Gradually, however, we find the author gliding into another phraseology, which is indeed the best adapted to express the phenomena of counter-irritation and the means by which it is performed, as when he speaks of "the application of stimulant agents," "a local stimulant treatment," "stimulants employed," etc. The writer's treatment should mean the treatment by irritants, whereas in the pages now before us it is represented to be carried out by stimulants. "The influence of diffusible stimulants in scattering, as it were, the earliest tendencies to internal congestion and inflammation, is familiarly exhibited in the effects of alcohol and heat upon persons benumbed with cold and beginning to suffer cold in some internal organs." It is true that immediately after this we read of external heat as belonging to the class of irritant medicines. Surely that degree of heat which is proper and grateful in such circumstances is anything but irritant—it is a mild stimulant. The first of these designations can only be applicable to an extreme degree of heat, either dry or moist.

Dr. Stillé seems, in making out the class of irritants and in his reasoning on irritant medication, to have followed pretty closely MM. Trousseau and Pidoux, (*Traité de Therapeutique et de Matière Medicale,*) who divide the subject into four sections, viz., the substitutive or homœopathic, the transpositive, the spoliative, and the exciting. How very arbitrary is one of these several terms to designate the action of irritants will be at once apparent after we shall have given the definition of spoliative by these writers, in connection with irritant medication. "There is spoliation, in the therapeutical sense, whenever the blood is made to part with one or more of its elements in proportion greater than occurs in the normal movement of organic composition and decomposition." According to this showing, then, blood-letting, purgatives, diaphoretics, and diuretics, are spoliatives; but do they come under the head of a separate class called irritants? MM. Trousseau and Pidoux restrict, however, the terms spoliative medication to that which is brought about by means of suppuration—a narrow restriction to a word of such large meaning. So, also, we would ask, whether "exciting medication" is not a treatment by stimulants rather than by irritants? In fine, there cannot but be continual embarrassment in attempting to explain, as possessed of analogous properties, the therapeutical operation of substances of so contrasted a character as irritants proper, whose primary action is displayed almost entirely on the skin, on which it produces high irritation and disorganizing inflammation, and a large number of stimulants, some tonics, and a few refrigerants which are taken into the stomach, and through it act on the whole animal economy in modifying excitement and nutrition by safe, moderate, and easy processes. The title of any class of medicines, if intended to indicate their general attributes as curative agents, can only be properly deduced from their primary operation and subsequently ascertained effects when given in ordinary medicinal doses. If we refuse this standard, we should be obliged to designate a great majority of the articles of the materia medica as stimulants approaching to irritants in the first instance. If we adopt it, as is virtually done in most of the systems of classification, we must discard from the class of irritants in the work before us acetic acid as readily as we would citric and the vegetable acids generally, and place them, as Dr. Wood has done, under the head of refrigerants. The dietetic value and the use of acetic acid in febrile and other excited states of the organism, as laid down by Dr. Stillé himself, are in direct antagonism to effects which might be supposed to follow irritants. Even in its external application, when much diluted, it lowers the temperature of the body and quiets agitation in typhus and scarlatina. We would put in a similar claim, to some extent, in favor of the mineral acids, especially the sulphuric when largely diluted. Their more general use in various diseases places them under the head of tonics, and, in reference to the sulphuric acid, of astringents also. Although we may be unable to classify satisfactorily the fixed alkalies, we have no hesitation in withdrawing them from the company of irritants, with which they are ranked by the author, whose statement of their therapeutical effects, corresponding with the usually received ones, is not at all in harmony with the primary attributes which he assigns them. Better claim may be set up in favor of ammonia as an irritant, whether we regard its local operation when applied either in the form of vapor or mixed with water, or its general action. The mineral salts of silver, copper, and zinc in ordinary medicinal doses are essentially tonic, and are, we think, out of place in the class of irritants. The most favorable examples of this affinity are found in the vegetable and animal kingdoms; mustard and capsicum representing the first, and cantharides the second.

Passing to the next class, that of Tonics, we find them subdivided into specific, simple bitter, and stimulant tonics, followed by Tonic and Aromatic stmulants. Under the head of specific tonics most space is deservedly given to Iron, the therapeutical action and diversified remedial powers of which are succinctly yet clearly stated by the author, who investigates, at the same time, the relative efficiency of the several preparations of this metal. The entire section on the subject merits a careful perusal and study, so that the reader may make its contents his own. The same praise is well merited for the next section, on Cinchona and its preparations.* Did our space permit, we should like to justify our opinion by copious extracts—but we must pass on, if we hope even only to indicate the prominent traits of the entire work. Our object from the beginning has been to direct the attention of our readers to its unquestionable value, with a view of inducing them to study it for their own behoof, rather than for us to attempt to give a miniature likeness or to

* Dr. Stillé, in quoting the writers who believe in the sedative operation of cinchona and its salts, has omitted Dr. Bell, who, in his published lectures on the practice of physic in connection with those of Dr. Stokes, 1840, denies that quinia can properly be called either a tonic or a stimulant, but that it is to all intents and purposes a sedative, and as such displays its curative powers in periodical fevers in which irritation is the predominant pathological state of the organism. (See Stokes and Bell, Lectures, first edition, in one volume, p. 592.) Dr. Dickson, in writing on the subject, thinks that the priority of view of the sedative operation of quinine is due to Dr. Bell.

make our gloss a substitute for the outpouring lessons of the erudite author. If occasionally, in traveling over its pages, we use the pen critical, it is in the same spirit and to the same purport in which we would hold discourse with him in his or our own study; feeling all the while that, even if our criticisms be correct, his treatise has body, and strength, and life to bear any pickings or even jerks to which we or others may subject it.

The subject of General Stimulants, which constitute the fifth class in the present work, is agreeably and tersely introduced in language which we had marked for insertion, but our waning space compels us to change our purpose. A reference to the volume itself will enable the reader, after perusing these explanatory remarks, to appreciate the difficulty of classification, at the same time that he will find arguments by the author himself, especially on the *modus operandi* of heat and of electricity, directly bearing us out in the criticisms which we offered on his application of the term irritants to what we regard as really stimulants.

First among the general stimulants whose effects are described in the present volume comes "Calor—Heat." "The term heat is used as a synonym of *caloric*, and is also applied to the sensation which caloric produces. Thus it stands both for cause and effect. Nor is this double application of the term attended with inconvenience in ordinary language, for we know nothing of caloric except through the phenomena of heat." The first division is of dry heat, in which are considered "the mode of action and the phenomena of radiated, solar, and artificial heat, of hot, dry air, and of heated solids." Notice is taken of the hygienic influences of insolation; and the physiological and therapeutical effects of dry air are illustrated by references to various experimental observations on the subject. Among the diseases benefited by this latter agency, are rheumatism, and, in a very marked manner, wounds. Dr. Guyot, after experiments on animals, and observing the influence of the remedy on the cicatrization of wounds of hospital patients after operation, draws the conclusion "that wounds without any dressing heal more rapidly in air of a temperature of 86° Fahrenheit than at a lower temperature, without dressing." Having spoken of the remedial action of the actual cautery, he describes that of *Mayer's hammer*, as of an analogous character. It occasions, after being plunged into boiling water and then applied to the skin, acute pain and an eschar. At a temperature of 130° there is permanent rubefaction; and at 120° the rubefaction continues only about an hour.

Under the head "Heat Combined with Watery Vapor," we read a description of vapor baths, general and local, and of their use in disease. The author errs somewhat when speaking of the fashion of vapor bathing "in Eastern countries:" his description applies to that of the people of the

North, more particularly the Russians. The succession of benches from below upward is found in the latter but not in the former. Turkish warm and hot bathing consists for the most part of exposure to a heated air of gradually increasing temperature, in which the bather is placed while passing from one room or hall to another; the process being completed by friction, shampooing, and the affusion or washing with warm or cold water, as may be most agreeable to the bather. The manipulations just mentioned are not, with the exception of strigillation, accessories peculiar to the Russian vapor bath. The Egyptians, says Prosper Alpenus, as quoted by Dr. Bell,* "visit the bath to sweat, to be rubbed, and to be washed, three processes gone through in different apartments and with different appliances." When at the bath, or rather bath rooms, the Egyptians, on the same authority, eat and drink, and take physic in its various modes.

A more regretable error occurs in Dr. Stillé's confounding the warm with the hot water bath, as has been done by some of our best medical writers—a fact attributable, in a great degree, to the irregular and imperfect use of bathing in general, either as an agent of hygiene or as a remedy for the cure of disease. "*Warm* water," we are told by the author, "is most usually employed as a bath at about 106° Fahrenheit." Water, we affirm, when used of this temperature, is unmistakably *hot*, and becomes a powerful stimulant, exciting both the vascular and nervous systems; and as such it is only applicable to states of torpor and inertia, cold skin and languid circulation, and to persons of a phlegmatic temperament. The warm bath proper, varying from 92° to 98°, is, on the contrary, soothing and tranquilizing, and for the most part inclines to sleep. Its stimulant power is slight, and it might rather be called a sedative, in its lower degree. Its reputed invigorating effect is owing to its abatement and removal of irritation and slight febrile excitement left by fatiguing travel or prolonged exercise. Very different from this are the effects of the author's warm bath, which he speaks of as "at a temperature of 100° to 110°," and which is in fact not only a hot, but a very hot bath, wholly inapplicable and contra-indicated under all the circumstances in which a legitimate warm bath is proper and useful. Again, the standard changes in the hands of the author, who tells us that chronic diseases of the skin, particularly of the scaly forms, are benefited by prolonged warm baths (at 96° to 100° Fahrenheit.) We are obliged, also, to criticise the definition furnished by the author, in degrees of the thermometer, of the tepid or lukewarm bath, which he makes to range from 85° to 95° Fahrenheit, and which is an encroachment on the limits of the warm bath

* Baths, etc., p. 149.

of three degrees. There are, it must be acknowledged, difficulties in affixing precise limits to the different divisions of baths, more particularly the cold and the tepid, but there may be, with a little care and observation, some approach to a standard, based on sensatio n, which will be generally recognized. In one, the upper direction, is "a boundary at which all difference of opinion depending on feeling ceases, it my be called that of unvariableness of temperature, and corresponds with the animal heat. This last is, with slight differences, everywhere the same; and a watery fluid of this temperature will impress every person immersed in it in very nearly a similar manner. A bath of 95° to 97° Fahrenheit will be called warm, both by the Laplander and the intertropical African."* In the opposite and lower direction, there will also be a common standard derived from sameness of sensation of coldness, experienced by those who use a bath under 70° Fahrenheit. "The shock will be evident, and there can be no hesitation in our designating it cold," although it will not be of that persistent nature which would be caused by water of a lower temperature, and it will be followed much more promptly by reaction than in the latter case. In a practical point of view, the difference between the tepid, the warm, and the hot bath, in their therapeutical action, are analogous to those between water, weak brandy-water, and the raw liquor itself.

The subject of "Electricity," the next general stimulant, is brought up, as might be expected, from the reading of the author, to the knowledge of the day. The various forms in which mechanical electricity is used are specified, and the mode of deportment, physiological action of galvanic or voltaic electricity, and induction are described. So also, magnetic electricity and induction electricity are explained. This is followed by an explanation of the modes of employing voltaic and magnetic electricity, and an enumeration of morbid conditions in which it is employed. The entire section has more freshness and fullness of detail than is met with in systematic courses on therapeutics and materia medica.

The next of the general stimulants is "Vinum—Wine." One might ask why not take up alcohol first, as representing the potential as well as medicinal and poisonous element in both wine and all fermented, as well as distilled liquors, and then treat of them in succession as subdivisions? The author has probably had in view, in making the existing division, the historical order in which the two classes of drinks—the vinous, which represents all fermented ones, and the alcoholic, which stands for ardent spirits—were brought to the notice and use of mankind. After a lucid notice of the history and dietetic use of wine, and of the various

* Bell on Baths, p. 163.

kinds now procurable for the table—their manufacture, constituents, and action on man—their uses are described in a therapeutical point of view.

"Alcohol" is discussed in a similar manner, but with more minuteness of inquiry into its toxical effects as an inebriating agent, and its possible or probable use as an article of hygiene. The phenomena of primary and occasional intoxication, and the effects of intoxication, are fully described. The author does not advocate the habitual use of alcoholic liquors. How indeed can we recommend, or even tolerate, as far as silence on the subject goes, the habitual use of a liquid which consists, in part, of one of the most active poisons known? There is no analogical reasoning to bear us out by reference to the composition of the various kinds of food habitually used by man for his nourishment. The use of alcoholic liquors as a beverage, without, in all cases, direct disease or manifest deterioration of bodily and mental vigor ensuing, is only an evidence, among others, of the wonderful powers of the human organism in tolerating and resisting deleterious and poisonous agents; but it ought not to be received, for a moment, as an evidence of these liquors being endowed with any safe sanatory activity when habitually used. There is no condition of the animal economy, either strength of body or of mind, endurance of heat and of cold, the most exhausting labors and exposures by sea and land, bravery in the field of battle, wisdom in council, the sublimest exhibitions of genius in every department of science and the arts, and the noblest creations of the imaginative faculty, but what have been evinced without any aid from the spurious inspiration of the juice of the grape or the alcoholic products of the still. Against the habitual use of alcoholic liquors of any description, though they should bring their alleged pleasures, stand the daily risk of perverted and disordered functions, and the constant danger of acquiring fixed and incurable habits of intoxication and all its dire concomitants and effects,—destructive not alone of the intellectual and moral character of the sufferer himself or herself—for woman is not exempt—but of the peace of mind and social position and means of living of relatives of the household, and not seldom of friends beyond it.

"Narcotics," furnishing subjects of very difficult handling, are treated by the author. The section on opium is both full and instructive, and is of itself enough to insure a favorable reception of the entire volume, even were its other contents of an inferior character, instead of being as rich and varied as we have described them. Narcotics in Dr. Stillé's arrangement come first under the sixth class, or "Cerebro-Spinal Stimulants"—Antispasmodics constitute the second division. In this are included the history of the origin and extensive application of etherization and of chloroform—subjects of great moment to practitioners in all the departments of medicine and surgery.—Spinants, (Tetanica,) the seventh class, are represented by Nux Vomica, Strychnia, and Faba Sancti Ignatii. Classes eighth, ninth, and tenth represent successively, general sedatives, arterial sedatives and nervous sedatives. The most important among the general sedatives, and that treated most in detail, is "Frigus—Cold." The history of opinions and of the therapeutic uses of this powerful agent are brought out quite distinctly by the author, whose inferences on some points we might contest, were opportunity allowed us for stating our reasons of dissent. Dr. Stillé's usual accuracy of reference to an author's work is at fault in the instance of the treatise of one of his own countrymen and fellow-citizens. We find at the foot of page 256, among other references, "Bell on the Watery Regimen." The real and known title of Dr Bell's volume* is "A Treatise on Baths, including cold, sea, warm, hot, vapor, gas and mud baths. Also, on the watery regimen, hydropathy, and pulmonary inhalations, etc."

Notwithstanding the opinion of Dr. Stillé, that "diluted hydrocyanic acid," another general sedative, "has just claims to confidence in the treatment of several diseases," and the corroborative testimony which he subsequently furnishes, we lean to the belief of MM. Trousseau and Pidoux, that this medicine "is often dangerous, almost always useless, and very rarely curative." Palliative and soothing it may be, but rarely shall we find it exert a positively controlling and permanent influence over an established disease. Its volatile nature and the great danger attending its use in doses beyond a very low standard, and from difference in recognized formulæ, ought to be balanced by very decided therapeutic powers. At the most, it is of some service in certain forms of neuralgia.

Among the "Arterial Sedatives," the only examples adduced in the work before us are Digitalis and Veratrum Viride, both of them modern, and the latter of the two a recent remedy. We object to the appositeness of the title as conveying too limited an idea of the physiological as well as therapeutical effects of the articles classed under this head. Attention must be directed to the very capricious and irregular action of digitalis, which was at first supposed to be antiphlogistic and a substitute for venesection and other evacuants, and is now thought to be best adapted to cases of feeble action or reduced excitement. Its most tranquilizing operation on the circulatory apparatus is on the heart much more obviously than on the arteries, and primarily through the nervous system. The same claims have been set up for veratrum viride as were at first urged in favor of digitalis, and experience will, we think, lead to the same conclusions in regard to the real pathological state removed or relieved by the former as have been reached already in the case of the

* First edition.

latter. No mention is made by Dr. Stillé of antimonial preparations, and especially tartar emetic as an arterial sedative. We had long taught, and practiced in the belief, that tartar emetic had considerable reducing powers as a direct sedative or contra-stimulant in various phlegmasiæ—before Dr. Wood had received it in his course of materia medica as an arterial sedative. The question is fully examined by the author under the head of Emetics, when speaking of antimonii et potassæ tartras.

The class of "Nervous Sedatives" includes medicines of great powers, two of which, Aconite and Veratrum Sabadilla, have come into large use of late years; while another, Conium, does not enjoy the vogue that it did in the last century. Tobacco, which is in this class, affords another example analogous to that already pointed out in the instance of alcohol, of one of the most active and speedily fatal of all poisons being habitually used by vast numbers of the inhabitants of our globe for its power of producing luxurious enjoyment and for its alleged hygienic efficacy. Such is its extreme activity of action on the human organism that we cannot be said to have yet obtained any satisfactory control over it as a therapeutic agent, except in an exceedingly small number of diseases. In others, as in strangulated hernia, its administration has been at different times attended with fatal consequences.

We cannot follow the author in the next great class of "Evacuants," and their subdivisions into those which act on particular organs or organic systems. Many nice questions in therapeutics come up for discussion and elucidation in speaking, for example, of emetics, cathartics, diuretics, diaphoretics, expectorants, and emmenagogues, and many interesting specifications of particular operation and curative powers of individual articles under the several divisions just named. We miss in the list of purgatives calomel, which only finds a place in the last class of the series, or that of alteratives. The omission is a great one, and for which we find no explanation.

Fine themes for discussion are afforded in the class of alteratives, each of the first three in which—mercury, arsenic, and iodine—would furnish material for a separate volume.

We need not sum up the opinions which we have expressed, in different parts of this review, of the undoubted merits of Dr. Stillé's treatise. It is one which ought to be in the possession of every practicing physician who himself has no time for extensive reading, but who, nevertheless, ought to be acquainted with the current views entertained, and positive conclusions reached, respecting the therapeutical, we might say available value of each and every article of the materia medica, as determined by clinical experience. If we were to entertain a wish for something additional to the obvious riches of the work, it would be a more frequent and decided expression of the author's own views. At the same time, it must be admitted that reticence in this respect is far preferable to the assumption of superior knowledge and the dogmatism of imaginary experience, which, we fear, is sometimes obtruded on us by writers who seem, while thus speculating beyond measure on the credulity of their readers, to forget entirely the sacred obligations under which they are placed, the moment they take on themselves the office of teacher in whatever relates to the health and cure of diseases of their fellow-creatures. Life is too valuable to be made a stake for games of paradox and the fitful suggestions of imagination.

